# Deep Learning Approach to Automatize TMTV Calculations Regardless of Segmentation Methodology for Major FDG-Avid Lymphomas

**DOI:** 10.3390/diagnostics12020417

**Published:** 2022-02-06

**Authors:** Wendy Revailler, Anne Ségolène Cottereau, Cedric Rossi, Rudy Noyelle, Thomas Trouillard, Franck Morschhauser, Olivier Casasnovas, Catherine Thieblemont, Steven Le Gouill, Marc André, Herve Ghesquieres, Romain Ricci, Michel Meignan, Salim Kanoun

**Affiliations:** 1 Centre de Recherche Clinique de Toulouse, Team 9, 31100 Toulouse, France; wendyrevailler@yahoo.fr (W.R.); thomas.trouillard@hotmail.fr (T.T.); 2 Institut Universitaire du Cancer de Toulouse, Institut Claudius Regaud, Nuclear Medicine, 1 avenue Joliot Curie, 31000 Toulouse, France; 3 Assistance Publique-Hôpitaux de Paris, Hôpital Cochin, Nuclear Medecine, René Descartes University, 75014 Paris, France; annesegolene.cottereau@aphp.fr; 4 CHU Dijon, Hematology, 10 Boulevard Maréchal De Lattre De Tassigny, 21000 Dijon, France; cedric.rossi@chu-dijon.fr (C.R.); olivier.casasnovas@chu-dijon.fr (O.C.); 5 Thales Services, 31400 Toulouse, France; rudy.iaaa@gmail.com; 6 ULR 7365—GRITA—Groupe de Recherche sur les formes Injectables et les Technologies Associées, University of Lille, CHU Lille, 59000 Lille, France; franck.morschhauser@chru-lille.fr; 7 Hemato-Oncology Unit, Saint-Louis University Hospital Center, Public Hospital Network of Paris, 75010 Paris, France; catherine.thieblemont@aphp.fr; 8 Department of Hematology, Nantes University Hospital, INSERM CRCINA Nantes-Angers, NeXT Université de Nantes, 44000 Nantes, France; steven.legouill@chu-nantes.fr; 9 Department of Hematology, Université catholique de Louvain, CHU UcL Namur, 5530 Yvoir, Belgium; marc.andre@uclouvain.be; 10 Department of Hematology, Hôpital Lyon Sud, Hospices Civils de Lyon, 69310 Pierre-Bénite, France; herve.ghesquieres@chu-lyon.fr; 11 LYSARC, Centre Hospitalier Lyon-Sud, 165 Chemin du Grand Revoyet Bâtiment 2D, 69310 Pierre-Bénite, France; romain.ricci@lysarc.org; 12 LYSA Imaging, Henri Mondor University Hospital, AP-HP, University Paris East, 94000 Créteil, France; michel.meignan-ext@aphp.fr

**Keywords:** total metabolic tumor volume, lymphoma, deep learning, convolutional neural network

## Abstract

The total metabolic tumor volume (TMTV) is a new prognostic factor in lymphomas that could benefit from automation with deep learning convolutional neural networks (CNN). Manual TMTV segmentations of 1218 baseline 18FDG-PET/CT have been used for training. A 3D V-NET model has been trained to generate segmentations with soft dice loss. Ground truth segmentation has been generated using a combination of different thresholds (TMTVprob), applied to the manual region of interest (Otsu, relative 41% and SUV 2.5 and 4 cutoffs). In total, 407 and 405 PET/CT were used for test and validation datasets, respectively. The training was completed in 93 h. In comparison with the TMTVprob, mean dice reached 0.84 in the training set, 0.84 in the validation set and 0.76 in the test set. The median dice scores for each TMTV methodology were 0.77, 0.70 and 0.90 for 41%, 2.5 and 4 cutoff, respectively. Differences in the median TMTV between manual and predicted TMTV were 32, 147 and 5 mL. Spearman’s correlations between manual and predicted TMTV were 0.92, 0.95 and 0.98. This generic deep learning model to compute TMTV in lymphomas can drastically reduce computation time of TMTV.

## 1. Introduction

The total metabolic tumor volume (TMTV) has recently been proposed as a tumor burden quantification method in various lymphoma subtypes, especially in Hodgkin lymphoma (HL) [1,2], diffuse large B cell lymphoma (DLBCL) [3] and follicular lymphoma (FL) [4]. This evaluation requires whole-body segmentation of the tumor mass on the baseline 18FDG PET/CT imaging. To make TMTV acceptable in routine clinical practice, a high level of automation is needed to reduce the calculation time and to enhance the interobserver reproducibility.

Such automatization approaches have been proposed using a large range of algorithms, and more recently, using deep learning segmentation algorithms [5,6,7]. Segmentation algorithms using convolutional neural networks (CNNs) have shown a very high accuracy for medical image segmentation [8,9]. These CNN approaches can exploit numbers of imaging features to distinguish tumoral uptakes from physiological uptakes such as brain, kidney or brown fat uptakes, which are common pitfalls in segmentation automation.

In the scope of TMTV calculations in lymphoma, many studies have demonstrate very high accuracy in predicting TMTV values and could be a major breakthrough for automating TMTV calculations [6,7,10]. However, published data are still limited in some respects. First, published papers usually rely on a single lymphoma subtype dataset, although it could be more interesting to use data of the most common lymphoma FDG-avid subtype to train a more generic model with increased dataset training (HL/DLBCL/FL having overlapping imaging pattern). Moreover, all those papers relied on a single TMTV calculation gold truth methodology, which seems to be a major limitation because TMTV methodology harmonization is still pending [11]. Training CNN models against a single segmentation methodology (ex: 41% SUVmax or 2.5 threshold) forces algorithms to try to reproduce one specific segmentation threshold, with a loss of control of the segmentation rule, which could not be transferred to other segmentation methodologies. Finally, the reported performance is still very dependent on the representativity of the training dataset and the quality of tumor labelling, making reported performances not always generalizable for routine applications.

One other approach for training CNN models could be to try to reproduce an equivalent of the manual physician delineation of uptakes, regardless of the final segmentation threshold algorithm. The final segmentation methodology would then be applied in a post-processing step to compute the final TMTV. With this approach, a more generalizable model could be built for TMTV calculations of FDG-avid lymphoma. 

The aim of our study was to train and validate a generic segmentation CNN model on a large training dataset to provide TMTV automation on the main lymphoma subtypes (DLBCL, FL and HL), regardless of the PET thresholding methodology.

## 2. Materials and Methods

### 2.1. Patients

The study population included 2030 baseline PET/CT of FDG-avid lymphomas—HL (n = 777), DLBCL (n = 851) and FL (n = 402)—collected from anonymized multicenter imaging trial datasets of the Lymphoma Study Association (LYSA): 703 for AHL2011 [12], 573 for GAINED [13], 277 for RELEVANCE [14], 217 for REMARC [3], 125 for FLIP [15], 61 LNH2007-3B [16] and 74 for PVAB [17] (Table 1).

Each of these studies has been approved by an ethics committee (see related publications); ancillary studies of images of these studies were planned from the initials study protocols.

### 2.2. Image Preprocessing

PET/CT quality was checked for slice interval regularity and axial slice completeness, and attenuation-corrected PET/CT images were converted into standardized uptake value (SUV) units.

All available PET, CT and ground truth masks followed a preprocessing pipeline. First, from the DICOM format, a 3D image in nifti (.nii) format was generated. Images were resized to a 128 × 128 × 256 and 4.0 × 4.0 × 4.0 mm voxel size with a linear interpolation. PET and CT were aligned at the same origin to build 4D PET/CT input data. Input data for training were scaled from 0 to 1 (corresponding to an original input range (−1000, 1000) for CT UH values and (0, 25) for PET SUV values). 

### 2.3. Ground Truth Generation

For each baseline PET/CT, ground truths were generated by the manual delineation of uptakes randomly assigned to 13 expert physicians trained in TMTV calculations from the LYSA group.

Manual regions of interest (ROIs) were drawn using the open-source PET/CT viewer for Fiji [18]. Four different thresholds were then applied to calculate the probability of the voxel being included in the TMTV segmentation: 41% SUVmax, SUV > 2.5 and SUV > 4.0 cutoffs and Otsu method (histogram-based threshold) [19,20,21].

A voxel-by-voxel average of these masks was computed to generate one single probability mask (probability from 0 to 1 with 0.25 steps), called the TMTV probability map (TMTVprob).

The rescaled PET/CT and the TMTVprob served as input data and ground truth for the CNN. 

### 2.4. Model Architecture and Training

The dataset was split into 60% for training (n = 1218), 20% for a test dataset (n = 407), and 20% was used for validation (n = 405).

A fully convolutional neural network VNET [22], with 4 levels and 8 channels in the first level, was trained on baseline PET-CT and TMTVprob, using Tensorflow 2.4.1 on a dual NVIDIA 1080 TI GPU. 

For each patient, a data augmentation strategy with random translating, rotation and scaling value was used to generate one additional augmented image. Model weights were updated using the stochastic gradient descent with a learning rate of 0.001 and momentum of 0.9, during 100 epochs. The soft dice loss was used as the loss function, defined as 1-dice_coefficient [22]. When we optimized our network through this function, we did not need to account for class imbalance between regions (background and tumors). The last layer had one channel and used the sigmoid activation function. 

### 2.5. Post-Processing

The predicted segmentation was converted to a raw TMTV prediction using a voxel probability threshold > 0.5. A post-processing step, consisting of applying a selected thresholding method, was then performed to reproduce three main different TMTV thresholding algorithms: 41% SUVmax, 2.5 and 4 SUV cutoff. 

For the 41% methodology, a clustering method was implemented to identify the SUVmax of individual lesions and compute the thresholded mask. This clustering was based on the isolation of connected components of the tumor mask prediction. Then, for remaining areas over 30 mL, a watershed segmentation was used to isolate tumor subparts using the SUV values of the PET image. After this clustering process, the relative threshold (41% SUVmax of each sub-component) was applied on each ROI. 

### 2.6. Predicted TMTV Validations

The raw TMTV predictions, before the post-processing step, were compared with TMTVprob and with the three TMTV manual delineations (41% SUVmax, SUV2.5 and SUV4) using the dice scores and Jaccard coefficients. 

Post-processed automated TMTV predictions using each thresholding algorithm (41% SUVmax, SUV2.5 and SUV4) were compared with the corresponding manual TMTV values, using the dice score, distribution analysis (max, min, mean, median, sd), Student’s *t*-test, Spearman’s correlation and Bland–Altman. 

## 3. Results

### 3.1. Training of the Convolutional Neural Network

The training was completed in 93 h on 1218 patients. In comparison to the TMTVprob, mean dice reached 0.836 in the training set, 0.835 in the validation set (405 patients) and 0.76 in the test set (407 patients with a median of 0.81). 

In the test set, the mean Jaccard coefficient was 0.64 ± 0.17, with a median of 0.68 and interquartile range (IQR) [0.55–0.76]. At the voxel level, the mean sensitivity (Se), specificity (Sp), positive predictive value (PPV), and negative predictive value (NPV) were 0.76 ± 0.17, 0.99 ± 0.00, 0.81 ± 0.18 and 0.99 ± 0.00, respectively. 

### 3.2. Raw TMTV Prediction without Post-Processing

Of the 407 patients of the test dataset, median dice scores of the raw predicted segmentation were 0.71 for 41%, 0.70 for 2.5 and 0.81 for 4 SUV cutoffs.

### 3.3. Clustering of Predicted Segmentation for 41% TMTV Calculation

The mean number of ROIs drawn by physicians during manual segmentation was 21 ± 20, with a median of 15.

Generated ROI number with watershed sub-segmentation of the whole body predicted mask was 33 ± 30, with a median of 25. 

### 3.4. Final TMTV Predicted Values Per Methodology

Of the 407 patients of the test dataset, median dice scores of the predicted segmentation were 0.77, 0.70 and 0.90 for 41%, 2.5 and 4 SUV cutoffs, respectively (Table 2).

**Figure 1:**
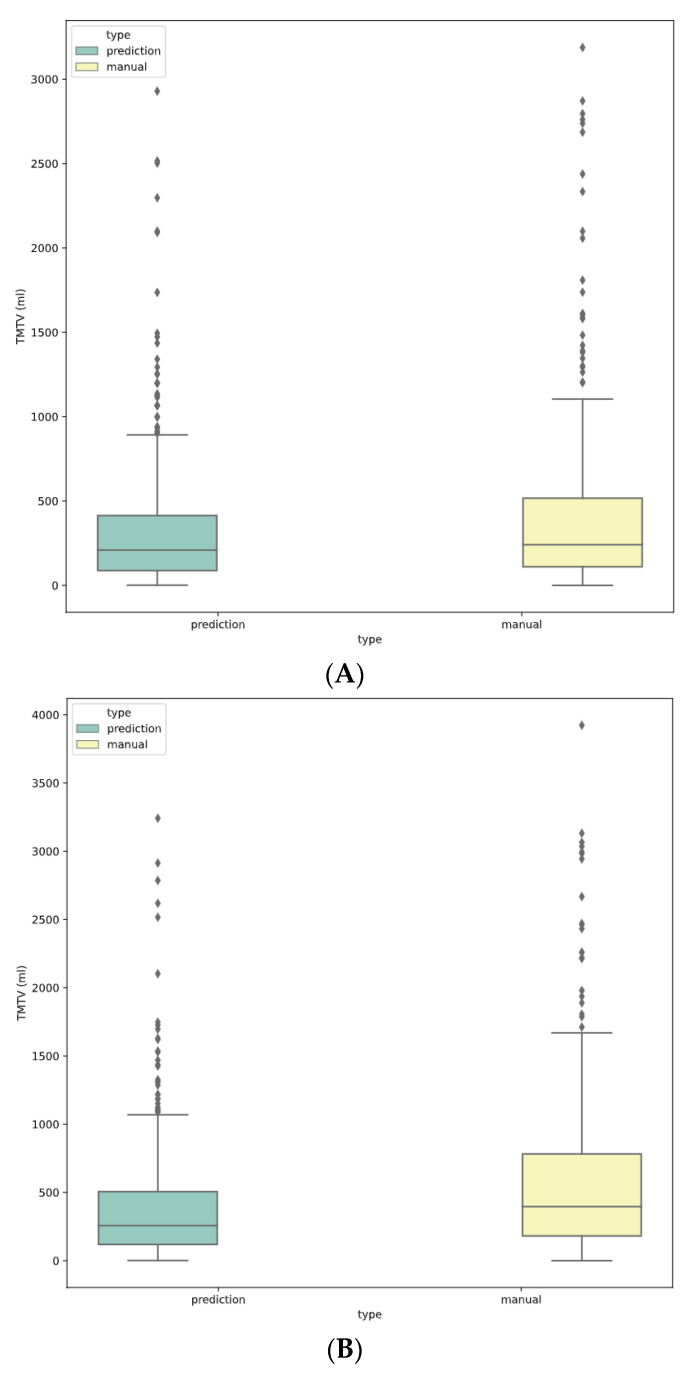

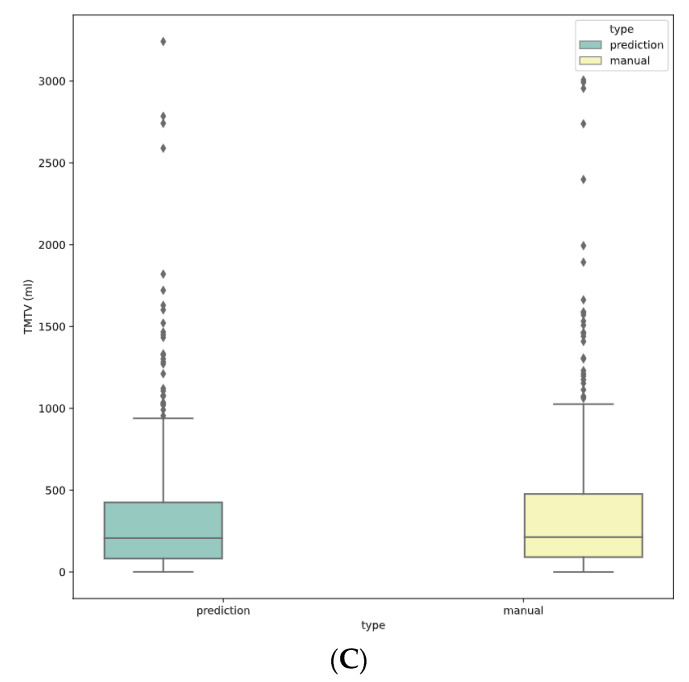
Boxplot of TMTV distribution for predicted and manual TMTV for each methodology (**A**) 41%, (**B**) 2.5, (**C**) 4.0.

**Figure 2:**
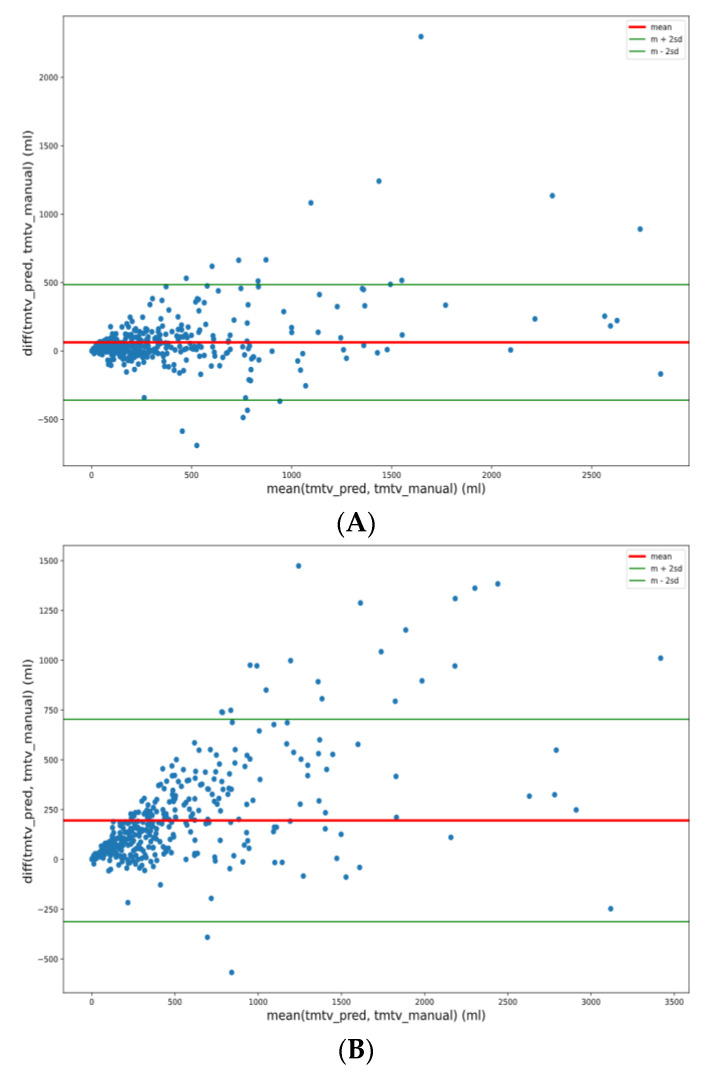

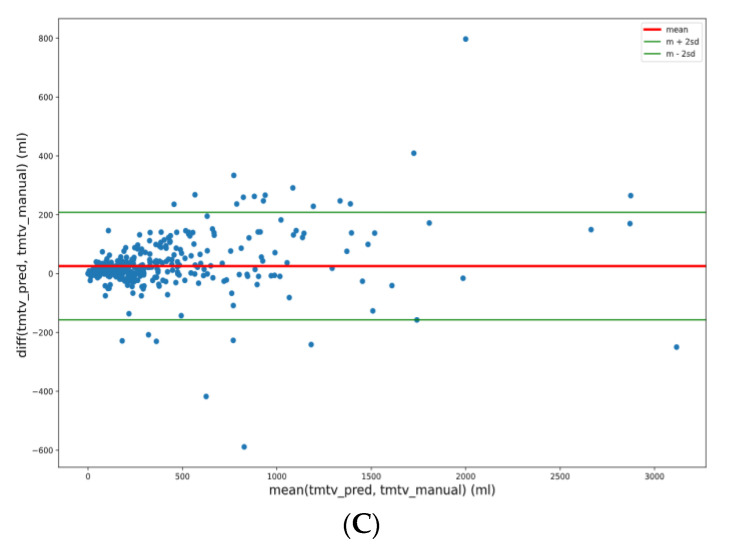
Bland–Altman plots between manual and predicted TMTV for each methodology (**A**) 41%, (**B**) 2.5, (**C**) 4.0.

Median TMTV values for manual and predicted segmentation were 240 ± 498 mL vs. 208 ± 420 mL for 41% SUVmax (NS), 400 ± 621 vs. 253 ± 472 mL for 2.5 SUV (*p* < 0.001), and 212 ± 457 vs. 207 ± 430 mL for 4 SUV cutoff (NS), respectively (Figure 1). Bland–Altman analyses are represented in Figure 2 for each methodology with limits of agreement and mean bias.

### 3.5. TMTV Correlation

**Figure 3:**
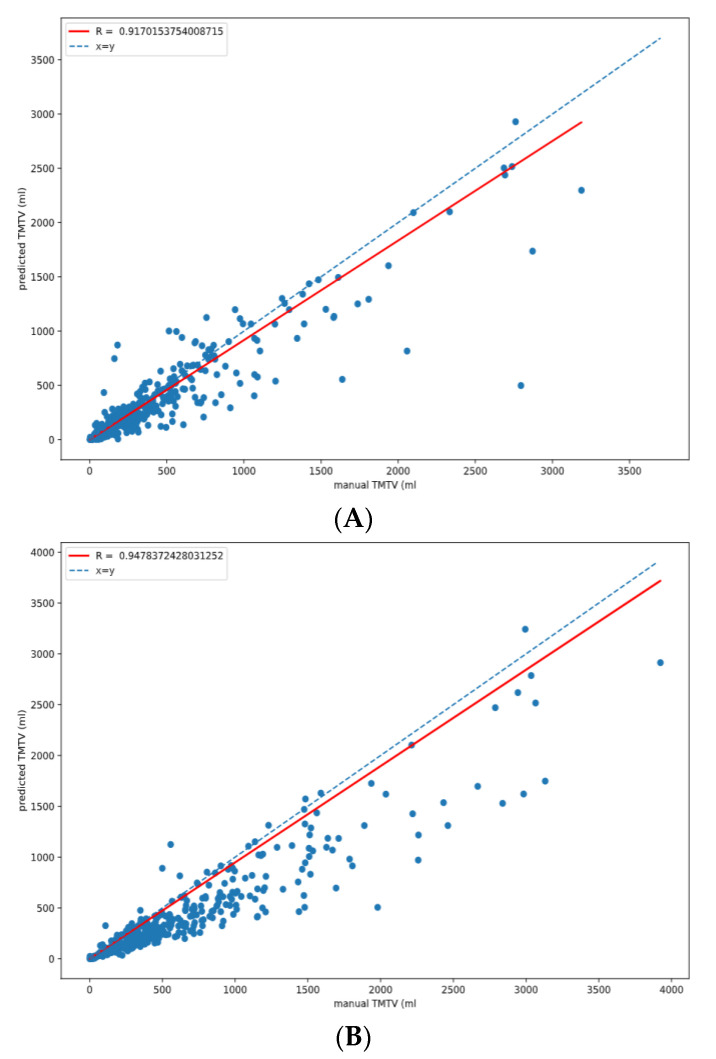

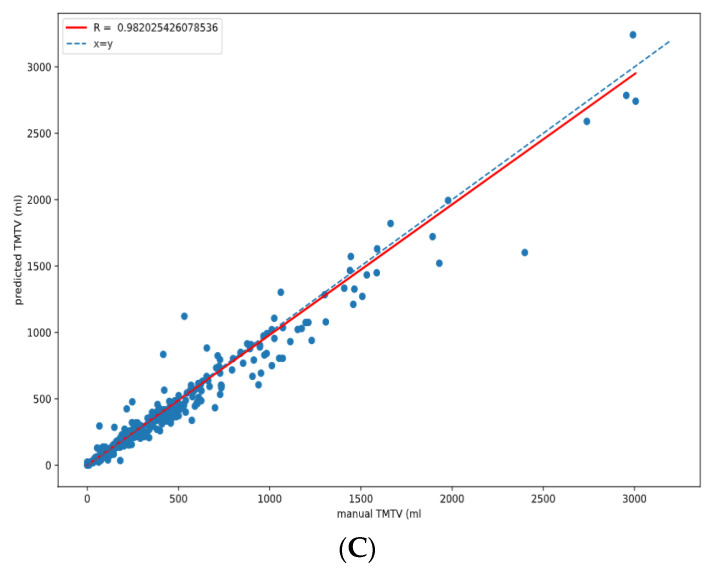
Correlation coefficient between the manual and predicted TMTV for each methodology (**A**) 41%, (**B**) 2.5, (**C**) 4.0.

The Spearman correlation coefficients with manual TMTV for 41%, 2.5 and 4 cutoffs were r = 0.92, 0.95 and 0.98, respectively, with *p* < 0.001 for each methodology. (Figure 3)

### 3.6. TMTV Predicted Values per Lymphoma Subtypes

Median dice scores for 41% SUVmax, 2.5 and 4.0 cutoff were 0.70, 0.68 and 0.93 for HL, 0.76, 0.68 and 0.90 for FL, and 0.85, 0.75 and 0.87 for DLBCL, respectively. (Table 2)

## 4. Discussion

TMTV calculations in various subtypes of lymphoma have recently become one of the most promising prognostic factors and may help to develop new risk-adapted treatment strategies [23]. Implementation of this new prognostic factor will require a high level of automatization to be routinely calculated.

Different approaches attempting to automatize TMTV calculation have been proposed to reduce the time-consuming delineation task to calculate tumor burden.

A first level of automation has been implemented in various medical views, such as region growing delineation [24], allowing single-click delineation, but still requires the manual identification of each target. Higher levels of automation have been proposed, such as component trees and connected operators [5], which are based on grey-level image intensity information and node hierarchies. This model enables whole-body segmentation without any input from the physician, but the result still lacks specificity, especially regarding physiological uptakes.

In recent studies, CNN architecture used cascaded networks, dividing the body into three different regions for the head, chest and abdomen, and then segmenting it using 2D or 3D CNN, as proposed by Jemaa et al. [10], or using direct 3D U-NET CNN on patched PET/CT, as proposed by Blanc-Durand et al. [7]. These proposed architectures rely on similar architecture to ours (V/U-NET); the difference is that the whole-body image is split into subparts to be able to fit the memory constraints of the GPU. A different approach has also been proposed by identifying FDG uptake with multi foci segmentation (MFS) over the image and then classifying them using CNNs to predict tumor vs. physiological uptake and localization, as proposed by Capobianco et al. [6]. In this case, the segmentation is defined by the MFS algorithm, which is an additional non-consensual delineation methodology that explains the reported correlation of 0.76 with manual TMTV 41%, which is fair but lower than the current study (r = 0.92 for TMTV 41%). 

All these CNN approaches achieved accurate segmentation with reported dice scores from 0.73 to 0.88, but relied on a single methodology for TMTV calculation; thus, they tried to reproduce a particular threshold although no consensual segmentation methodology has yet been defined. 

In contrast to cascaded networks, we chose to use a 3D V-NET with a whole-body image, resampled to a 4 mm isotropic cubic voxel included in a physical CNN-fixed input space. This kept the whole-body image (acquisition field up to 102 cm) without altering the image ratio, harmonized the image resolution of the wide range of image resolutions of the dataset, and was able to train the CNN with input fitting the GPU memory constraint. 

In the present study, the CNN was trained against a probability map of segmentation that reflected the segmentation methodology differences. These methodological differences are particularly significant in the edges of the uptake; thus, combining these different thresholds into a probability map forces the CNN to generalize a generic segmentation regardless of the thresholding cutoff. This choice also enables dissociation of the lesion detection from the segmentation algorithm, ensuring that the first level of uptake segmentation id generic enough to then be thresholded in a post-processing step to reproduce clinically validated TMTV segmentation algorithms. This two-step strategy is particularly important because it can merge automated TMTV delineation with manual TMTV delineation, as both techniques share the same final segmentation rule.

The achieved median dice score of the TMTVprob on the validation set was 0.81, which seems similar to the reported dice score in the literature, despite the methodological choice to reproduce a generic TMTV segmentation rather than a specific one. This segmentation accuracy is probably due to the large training dataset being the largest manually labelled dataset of baseline lymphoma PET/CT, to the best of our knowledge. 

Among the segmentation methodologies, the 41% SUVmax was the hardest to reproduce; in this methodology, the threshold is set per lesion, and thus needs to identify subparts of the whole-body segmentation inference. Although identifying non-connex segmented tumors is straightforward, lymphoma segmentation is also challenging because of large coalescent and heterogeneous uptakes. To solve this issue, we chose to sub-segment tumor masses over 30 mL, which are likely to be coalescent tumors, to isolate the subparts. For this lesion sub-segmentation, we chose the watershed algorithm which allows separation of regions of different uptake while maintaining a topographic surface approach: uptake zones of similar intensity are isolated in a continuous tumor unit in which the SUVmax-based threshold could be calculated. This algorithm enables the rough reproduction of manual segmentation from the binary-predicted whole-body segmentation and generates a comparable number of ROIs compared with manual segmentation.

In this study, we have shown a very strong correlation for each segmentation methodology (>0.9) and a relatively acceptable difference in median TMTV (CNN vs. manual). The demonstrated difference in median TMTV (5 to 147 mL) has to be interpreted regarding the high SD of the TMTV value (>400 mL). From these methodologies, the 2.5 SUV threshold appeared harder to reproduce with lower concordance values, probably because this threshold is too low and may include several background voxels which are discordant from other methodologies, and thus more difficult to include in a generic segmentation.

All of these results have been generated without any manual correction, highlighting the very good accuracy of CNN segmentations regardless of the methodology or lymphoma subtypes, because the three main FDG-avid lymphomas have shown similar performances.

**Figure 4:**
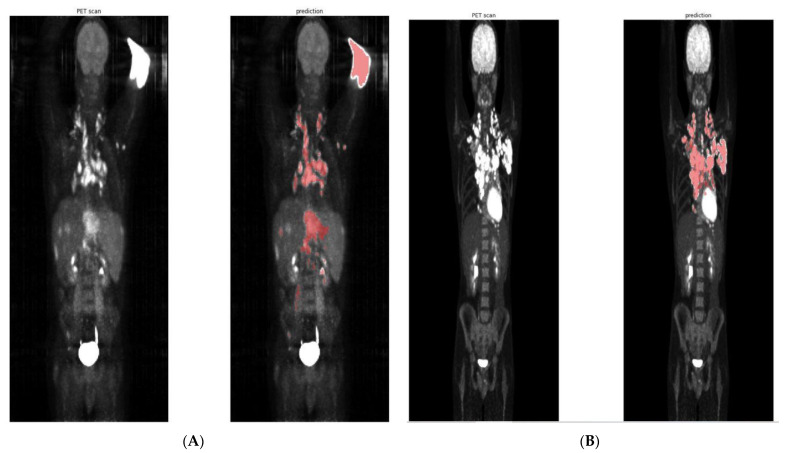
Predictions with false positives at the left arm FDG injection site, dice =0.32 (**A**) vs. accurate predictions, dice = 0.84 (**B**) of two different patients from the AHL cohort (HL).

In the perspective of using TMTV in decisional trials or in routine clinical practice, manual reviews and corrections will still be needed because the visual validation of segmentation still enables the removal of some obvious false-positives (e.g., tracer extravasation, ectopic kidney) (Figure 4) and to add false-negative lesions. All these segmentation corrections are limited and do not seem to introduce bias in a statistical point of view in this large cohort, but are still required at the individual patient level. 

The combination of a well-trained CNN model with a medical image viewer with segmentation features would bring TMTV calculation ease, reproducibility and accuracy to be implementable in routine clinical practice. For this purpose, we developed the free and open-source Dicom-To-CNN [25] library (MIT License) which allows DICOM preprocessing and exporting CNN outputs using interoperable DICOM formats (DICOM RTSTRUCT and/or DICOM SEG). Collaborations with major open-source medical image processing projects are currently in progress to develop the final integration of this deep learning segmentation algorithm including inference, visualization and editing of the segmentation output. Once complete, it will be possible to validate the feasibility of a routine use of TMTV in a real AI-guided medical workflow. This final validation will evaluate the global TMTV determination time and its inter-reader reproducibility to be integrated in decisional therapeutic strategies.

In conclusion, TMTV automation with deep learning algorithms have demonstrated very high performance, regardless of the segmentation threshold methodology in the main FDG-avid lymphoma subtype, making a generic segmentation model to be implemented in clinical trials evaluating TMTV-based treatment strategies. 

## Figures and Tables

**Table 1:** Ann Arbor stages in the patient population.

Original Dataset	Ann Arbor Stages
AHL2011	IIB, III, IV
GAINED	I–IV
RELEVANCE	I–IV
REMARC	II–IV
FLIP	I–IV
LNH2007-3B	I–IV
PVAB	II–IV

**Table 2:** Comparison between post-processed automated TMTV prediction and the corresponding manual TMTV values using dice coefficients for each methodology and lymphoma subtype.

Lymphoma Subtype		Dice Score per TMTV Segmentation Cutoff		
		41% SUVmax	2.5 SUV	4.0 SUV
HL	Median Mean ± SD	0.7 0.68 ± 0.16	0.68 0.67 ± 0.11	0.93 0.90 ± 0.10
FL	Median Mean ± SD	0.76 0.68 ± 0.22	0.68 0.64 ± 0.18	0.9 0.86 ± 0.17
DLBCL	Median Mean ± SD	0.85 0.79 ± 0.20	0.75 0.70 ± 0.19	0.87 0.82 ± 0.15
All Patients	Median Mean ± SD	0.77 0.73 ± 0.20	0.7 0.68 ± 0.16	0.9 0.86 ± 0.15

## Data Availability

Restrictions apply to the availability of these data. Original data (medical images) were obtained from the promoters of primary trials.
